# Influence of defects on the enhancement of thermoelectric properties in Sn-doped ZnO nanostructure synthesized via hydrothermal route

**DOI:** 10.3389/fchem.2025.1598509

**Published:** 2025-06-03

**Authors:** Danish Arif, Rajwali Khan, Adeel Younas Abid, Kashif Safeen, Adnan Ali, Mohammed A. Amin, Khizra Akram, Kamal Hussain Khan, Zulfiqar Ali, Akif Safeen

**Affiliations:** ^1^ Department of Physics, University of Poonch Rawalakot, Rawalakot, Pakistan; ^2^ National Water and Energy Center, United Arab Emirates University, Al Ain, United Arab Emirates; ^3^ Department of Physics, University of Sargodha, Sargodha, Pakistan; ^4^ Department of Physics, Abdul Wali Khan University Mardan, Mardan, Pakistan; ^5^ Department of Physics, Government College University, Faisalabad, Pakistan; ^6^ Department of Chemistry, College of Science, Taif University, Taif, Saudi Arabia; ^7^ Department of Physics, Women University of Azad Jammu and Kashmir Bagh, Azad Kashmir, Pakistan; ^8^ National Centre for Nanotechnology, Department of Metallurgy and Materials Engineering, Pakistan Institute of Engineering and Applied Sciences (PIEAS), Nilore, Islamabad, Pakistan

**Keywords:** ZnO, hydrothermal, doping, thermoelectric, sustainable energy

## Abstract

The efficiency of materials’ thermoelectric properties is often limited by various factors, and enhancing these properties through defect engineering is an effective strategy. This study investigated the defects-induced thermoelectric characteristics of Sn-doped ZnO nanoparticles. The samples were synthesized using the hydrothermal technique with varying concentrations of Sn. X-ray diffraction analysis confirmed that pure and Sn-doped ZnO nanoparticles exhibit a wurtzite structure, with an average crystallite size ranging from 22.8 to 18.1 nm. SEM micrographs revealed rod-like morphology which changes into spherical and irregular morphologies across all samples, with increased agglomeration observed with doping. EDX analysis verified the Sn incorporation into Sn-doped ZnO nanostructure. The photoluminescence (PL) spectrum showed significantly enhanced green emission, attributed to an increase in defect concentrations with doping. The electrical conductivity is increased with doping while the Seebeck coefficient reached the highest value of 166 μV/K for the SZ-2 sample, which is higher than any other synthesized sample. This behavior of the thermoelectric properties can be attributed to the presumable increased free carrier density induced by Sn doping in the ZnO crystal lattice, which enhanced both the Seebeck coefficient and electrical conductivity, thereby improving thermoelectric efficiency.

## 1 Introduction

The study of renewable energy has gained considerable focus over the past few years owing to the increasing need to meet global energy requirements. With a substantial portion of produced energy over 70% being wasted globally, addressing this inefficiency has become critical. Thermoelectric energy harvesting provides a viable approach by capturing waste heat from sources like vehicle exhausts and factory chimneys, contributing to improved energy utilization (Burnete et al., 2022; Miao et al., 2025). The growing global demand for energy production, conservation, and management has highlighted the importance of thermoelectric (TE) materials. These materials have emerged as a promising solution for both primary power generation and energy conservation, such as harvesting waste heat. The effectiveness of thermoelectric materials in addressing energy challenges lies in the efficiency of the thermoelectric module. These modules are solid-state devices that convert temperature gradients directly into electrical energy. They operate without the need for moving parts, produce no greenhouse gas emissions, and rely on electrons as their working medium (Liu et al., 2025; Wang et al., 2025). Thermoelectric materials, capable of directly converting thermal energy into electricity, have garnered considerable interest for their potential applications in sustainable and alternative energy systems (Singh et al., 2024; Massetti et al., 2021). The efficiency of these materials is commonly assessed using a dimensionless Figure of merit, ZT, expressed as ZT = σS^2^T/k, where S is the Seebeck coefficient, σ is electrical conductivity, and k is thermal conductivity (Al-Galiby et al., 2025; Wang et al., 2024). Thermal conductivity, indicating how well a material can transfer heat, is influenced by various factors and can be broadly divided into lattice and electronic contributions (Sarkar et al., 2024). These arise from interactions with phonons, lattice imperfections, impurities, electrons, grain boundaries, and interfaces. Strategies to reduce thermal conductivity include introducing structural defects such as point defects, dislocations, precipitates, engineered nanostructures, and applying thermal treatments to degrade the crystal quality of the material (Zheng et al., 2021; Chernatynskiy et al., 2018; Masoumi and Pakdel, 2022; Lu et al., 2025).

Recent research has focused on developing materials and techniques to achieve higher thermoelectric performance, with a ZT exceeding 2. High-temperature thermoelectric applications have extensively explored materials like SiGe alloys, rare-earth chalcogenides, and transition-metal selenides (Hasan et al., 2020). Currently, the most effective thermoelectric materials include metalloid alloys such as Bi_2_Te_3_ and PbTe, which are effective at medium and low temperatures (d’Angelo et al., 2023; Jamwal and Mehta, 2019). Since industrial processes generate significant high-temperature waste heat, thermoelectric modules that function efficiently at high temperatures are in demand. However, the high cost and oxidation susceptibility of these materials restricts their large-scale use (González‐Barrios et al., 2024). Oxides present a promising alternative due to their non-toxic nature, and excellent chemical and thermal stability. Both p-type and n-type thermoelectric materials are crucial for constructing a thermoelectric module, though progress in developing efficient n-type oxide materials lags behind that of p-type materials (Baskaran and Rajasekar, 2024). ZnO is an abundant, wide-band gap n-type semiconductor that has attracted significant attention due to its potential applications. It can simultaneously detect light and temperature signals through both photoelectric and thermoelectric effects (Wang et al., 2018; Sulaiman et al., 2022). Numerous approaches have been explored to improve performance of ZnO, including cationic substitutions with elements such as Al, Ga, In, Ti, Sb, and Co.; developing ZnO-based composites; improving material densification; and optimizing the structure of ZnO (Yin et al., 2023; Uzar and Abdulaziz, 2024; Ali et al., 2021). Extensive research on ZnO has focused on doping, including dual doping with group 3 and group 5 elements, as well as transition metals. The thermoelectric performance of ZnO is significantly influenced by defect concentrations, such as oxygen vacancies and zinc interstitials, however, it is only limitedly explored (Liu et al., 2021). Therefore, understanding the electrical properties of ZnO is crucial for improving its performance. To enhance its thermoelectric properties, doping and nanostructuring techniques have been explored. These approaches improve electrical conductivity and reduce thermal conductivity by promoting phonon scattering at grain boundaries, which is crucial for optimizing thermoelectric efficiency. Previous studies have shown that at room temperature, the peak ZT value of Ga-doped ZnO nanowires surpasses that of pure ZnO nanowires by a factor of 2.5 (Liu et al., 2021). In recent studies, Jood et al. reported a ZT of approximately 0.44 at 1000 K with thermal conductivity around 2 W/mK, though more enhancements are needed (Jood et al., 2011). Cd_1-x_Zn_x_O showed ZT of 0.52 at 1000 K with 1% doping (Prasad and Bhame, 2020). This study focuses on doping tin into ZnO, leveraging its ionic radius, which is similar to that of ZnO, making it likely to integrate well into the ZnO matrix. Tin also offers abundant electron states that can enhance carrier concentration, a crucial factor for optimizing TE performance.

The objective of this study is the strategic use of Sn doping to engineer defect levels within the ZnO lattice and their impact on thermoelectric performance. This research work provides a detailed understanding of how Sn substitution for Zn induces structural distortions and increases oxygen vacancy concentrations, which in turn modulate the electronic band structure. The findings reveal a significant improvement in the Seebeck coefficient and power factor with an increase in tin doping concentration, particularly at 1 wt%. The study offers new insights into the role of defect engineering in tuning the transport properties of ZnO, demonstrating that controlled defect engineering through doping is an effective pathway to enhance its thermoelectric efficiency.

## 2 Materials and methods

### 2.1 Materials

The synthesis of pure and Sn-doped ZnO involved the use of precursors such as zinc nitrate hexahydrate Zn(NO_3_)_2_⋅6H_2_O, tin nitrate hexahydrate Sn(NO_3_)_2_⋅6H_2_O, and sodium hydroxide (NaOH), all with 99.99% purity. These materials, sourced from Sigma Aldrich, were utilized without additional purification.

### 2.2 Material synthesis

**SCHEME 1:**
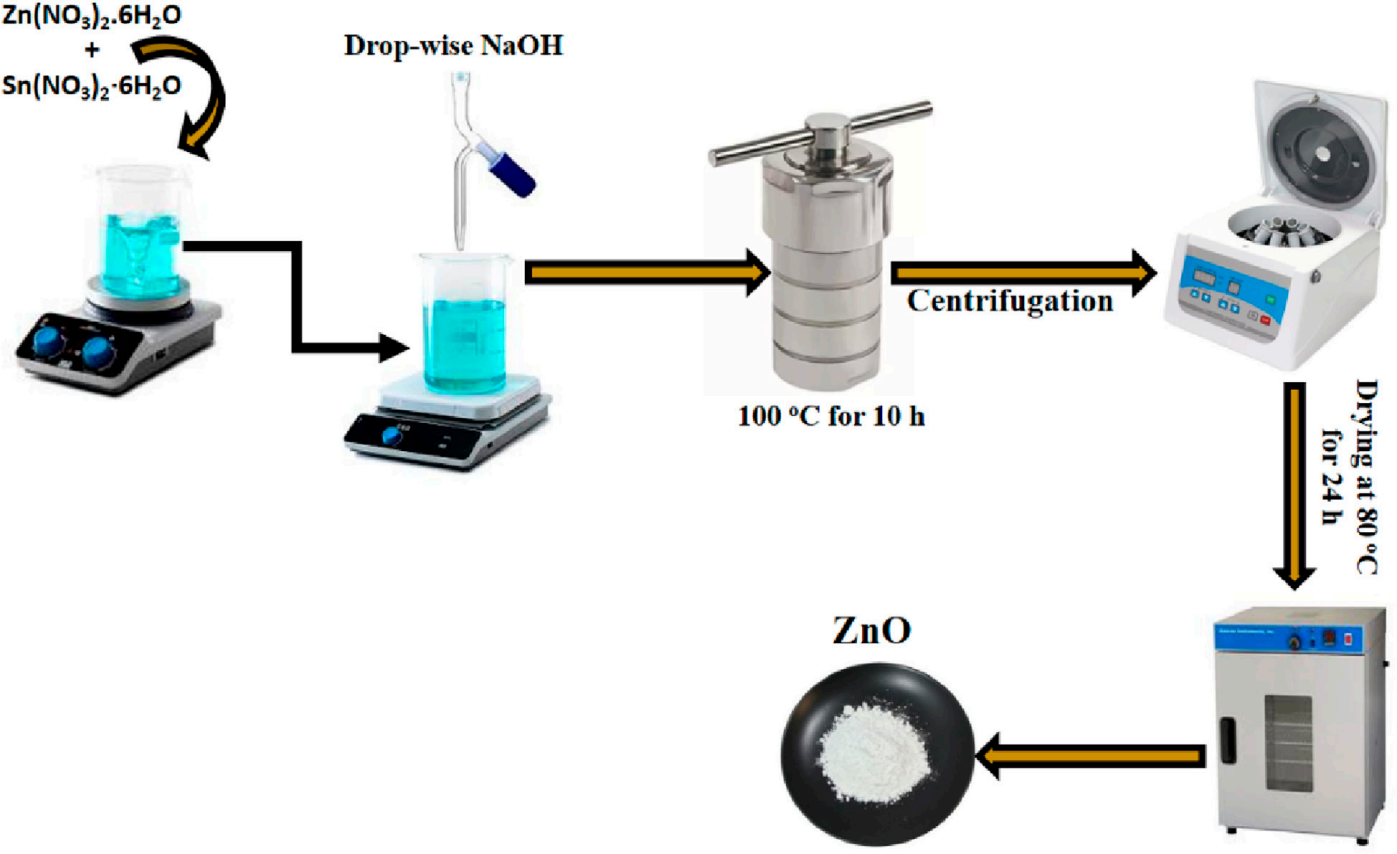
Hydrothermally synthesis of ZnO.

The study utilized a hydrothermal process to synthesize both undoped and Sn-doped ZnO powders. Sodium hydroxide (NaOH) served as the base for reacting with zinc nitrate hexahydrate. Two aqueous solutions, 0.4 M NaOH and 0.78 M zinc nitrate, were mixed at room temperature, resulting in a whitish precipitate at a pH of 10. This mixture was heated in an autoclave at 100°C for 10 h. The resultant precipitates were washed five times using a centrifuge with a methanol (20%) and distilled water (80%) solution to remove impurities. The cleaned product was dried at 80°C for 24 h and subsequently treated in a furnace at 600°C for 2 h to produce undoped ZnO powder. For Sn-doped ZnO, tin nitrate was added to the precursor solution, following the same synthesis steps as for undoped ZnO. The prepared samples were labeled as ZnO (pure ZnO), ZS-1 (0.5% Sn-doped ZnO), ZS-2 (1.0% Sn-doped ZnO) and ZS-3 for 1.5% Sn-doped ZnO. The synthesis of ZnO is given in Scheme 1 as follows;

### 2.3 Material characterizations

The structural, morphological, and optical features of undoped and Sn-doped ZnO nanoparticles were investigated using various analytical methods. Techniques such as X-ray diffraction (XRD) were employed to examine the crystalline structure, scanning electron microscopy (SEM) was utilized to observe surface morphology, and fluorescence spectrophotometry was applied to study optical properties. Furthermore, thermoelectric parameters, including electrical conductivity along with the Seebeck coefficient, were measured across a temperature range of 400°C–900°C using the NETZSCH SBA 458 Nemesis system.

## 3 Result and discussion

### 3.1 Structural study

**FIGURE 1:**
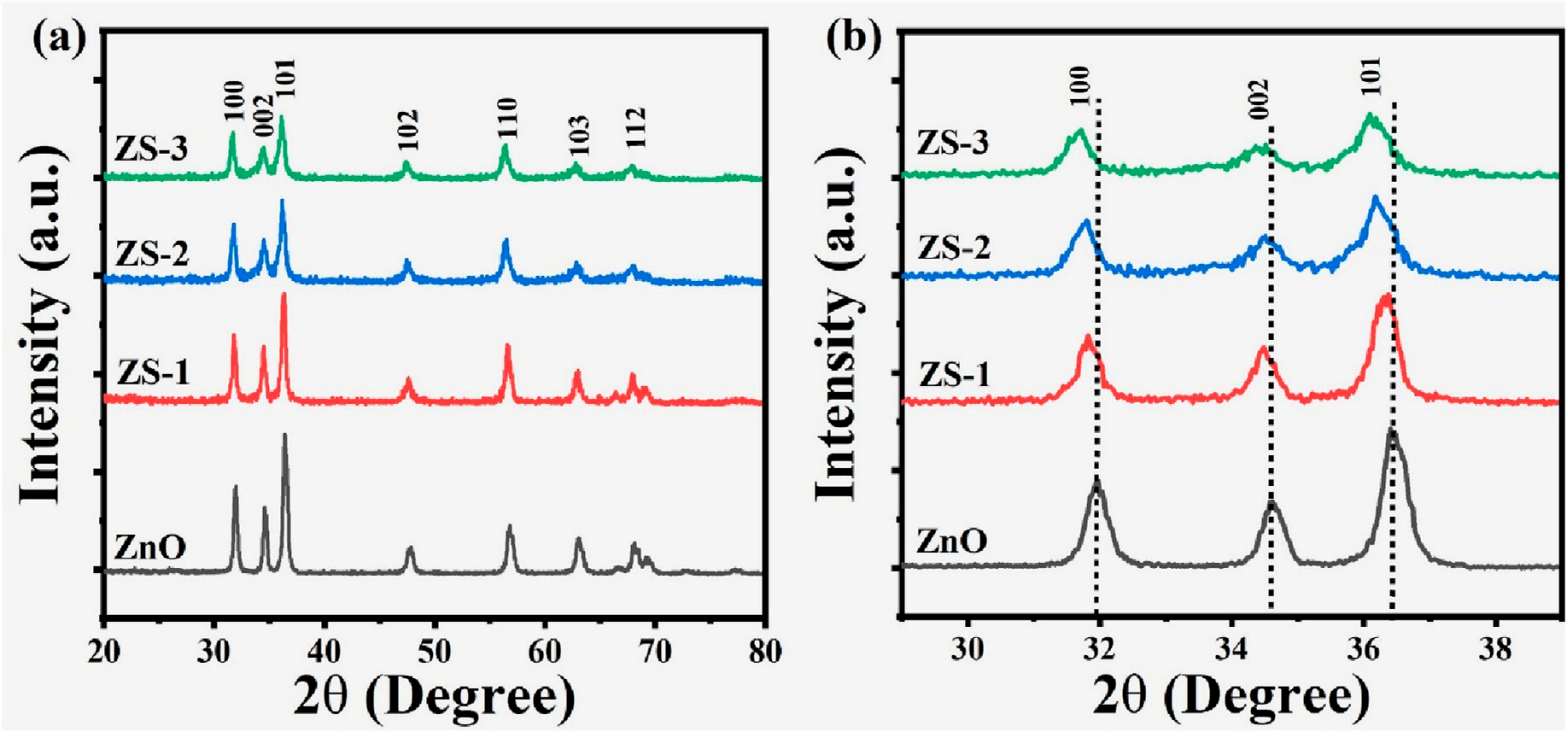
**(a)** XRD analysis of pure and Sn-doped ZnO; **(b)** Diffraction peak shift in Sn-doped ZnO.

The structural characteristics of both undoped and Sn-doped ZnO samples at varying concentrations of Sn were examined using X-ray Diffraction (XRD). The XRD patterns exhibited prominent peaks at 2θ angles of approximately 31.81°, 34.20°, 36.30°, 47.74°, 56.48°, and 63.10°, which correspond to the (100), (002), (101), (102), (110), (103), (201), and (200) crystallographic planes as shown in Figure 1a. No additional impurity peaks were observed in the patterns. These diffraction peaks are consistent with the hexagonal wurtzite structure of ZnO and align with the reference JCPDS card # 36–1,451. Figure 1b indicates that the peak position for the (001), (002), and (101) planes of Sn-doped ZnO shifts to a lower diffraction angle compared to pure ZnO indicating the presence of Sn. This shift can be attributed to changes in the intrinsic strain of the crystals. With an ionic radius of 0.069°nm, Sn^4+^ is smaller than Zn^2+^, which has a radius of 0.074 nm causing defects and lattice distortions within the crystal structure upon doping (Pan et al., 2013). The high intensity and sharpness of the peaks indicate a high degree of crystallinity. However, with the incorporation of Sn doping, the peak intensity decreases, suggesting a reduction in crystallinity (Safeen et al., 2023).

A slight increment in the full width at half maximum (FWHM) is detected in the doped samples, suggesting a decrement in crystalline quality. This may be due to dopant atoms not having favorable nucleation sites during crystal growth. The (101) lattice plane exhibits the highest intensity, signifying a preferred orientation in the crystalline structure.

Debye–Scherrer formula and Bragg’s equation are used for the determination of crystallite size and d-spacing as expressed in Equations 1, 2, respectively (Mubeen et al., 2023; Elboughdiri et al., 2023).

$$D=\frac{K\lambda }{\beta \mathrm{COS}\theta }$$ (1)

2d⁡sin⁡θ=nλ (2)

Here, in the above equations *λ, θ,* and *β* denote the wavelength of X-rays, diffraction angle, and FWHM, respectively. It can be seen that undoped ZnO exhibits higher crystallite size than Sn-doped ZnO and values are shown in Table 1. The lattice parameter and volume of the unit cell (V) of all the synthesized samples were calculated using Equations 3, 4, respectively, and their corresponding values are shown in Table 1.

$$\frac{1}{d^{2}}=\frac{4}{3\;}\;\frac{h^{2}+hk+k^{2}}{a^{2}}+\frac{l^{2}}{c^{2}}$$ (3)

$$V=\frac{\sqrt{3}}{2}\;a^{2}\times c$$ (4)

**TABLE 1:** Average crystallite size, lattice parameters, unit cell volume, d-spacing, and lattice strain of Undoped and Sn-doped ZnO.

Samples	Avg. Crystallite size (nm)	Lattice parameters		Unit cell volume (Å^3)^	*d*-spacing (nm)	Strain (ε) 10^−3^	Dislocation density (δ) (m-2)
		a = b (Å)	c (Å)				
ZnO	22.8	3.2432	5.2015	47.37	2.4651	1.0343	0.001923
ZS-1	21.5	3.2421	5.1993	47.32	2.4690	1.0734	0.002163
ZS-2	20.1	3.2401	5.1979	47.25	2.4733	1.2072	0.002475
ZS-3	18.1	3.2389	5.1967	47.21	2.4772	1.0704	0.003052

The symbols *h, k*, and *l* signify the Miller indices of the crystal planes, while the lattice parameters are denoted as *a, b*, and *c*. The parameter *d* refers to the interplanar spacing between two adjacent crystal planes. The lattice strain of undoped and Sn-doped ZnO nanopowder was calculated using Equation 5.

$$\text{strain }\left(\varepsilon \right)=\frac{\beta }{4\tan\theta }$$ (5)

The dislocation density (d), refer to the length of dislocation lines within a unit crystal volume, was determined using Equation 6 based on the Williamson and Smallman method (Akl and Elhadi, 2020; Mahmood et al., 2023). Higher values of crystallite size (D) and lower values of dislocation density (δ) reflect improved crystallinity of the nanoparticles. The analysis revealed that the dislocation density in the doped samples decreased with increasing Sn concentration. This trend indicates the formation of structural defects, as evidenced by photoluminescence spectra, which play a critical role in enhancing the thermoelectric properties of oxides.

$$\delta =\frac{1}{D^{2}}$$ (6)

### 3.2 Morphological and elemental study

**FIGURE 2:**
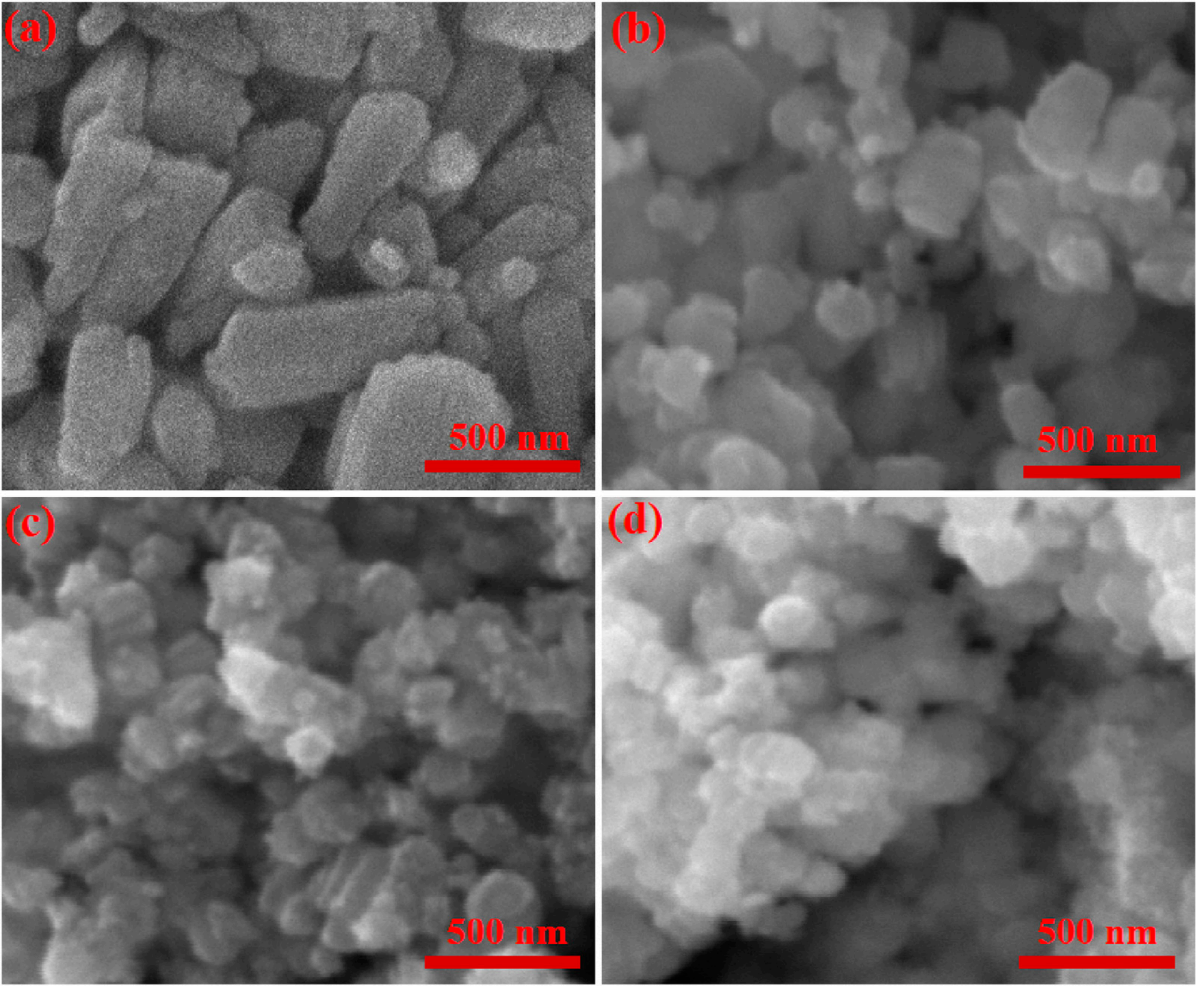
SEM micrograph of **(a)** ZnO **(b)** ZS-1 **(c)** ZS-2 **(d)** ZS-3.

Scanning Electron Microscopy (SEM) is a versatile tool used in various fields to analyze the surface morphology and structure of materials at the nanoscale. SEM micrographs of undoped and Sn-doped ZnO samples are depicted in Figure 2. Undoped ZnO consists of small rod of different size as shown in Figure 2a. ZS-1, ZS-2, and ZS-3 samples show spherical and irregular shape particles with doping agglomeration increased in all the samples as illustrated in Figures 2a–c. The transformation of ZnO morphology from rodlike to spherical or irregular shapes (Figure 2d) upon doping is primarily attributed to the influence of dopants on nucleation kinetics, crystal growth mechanisms, and surface energy dynamics during the synthesis process. The incorporation of dopants induces strain and structural defects within the ZnO lattice due to the size mismatch between the dopant ions and Zn^2+^ ions. These lattice distortions disrupt the preferred anisotropic growth direction, thereby promoting the formation of irregular morphologies and non-uniform particle shapes.

**FIGURE 3:**
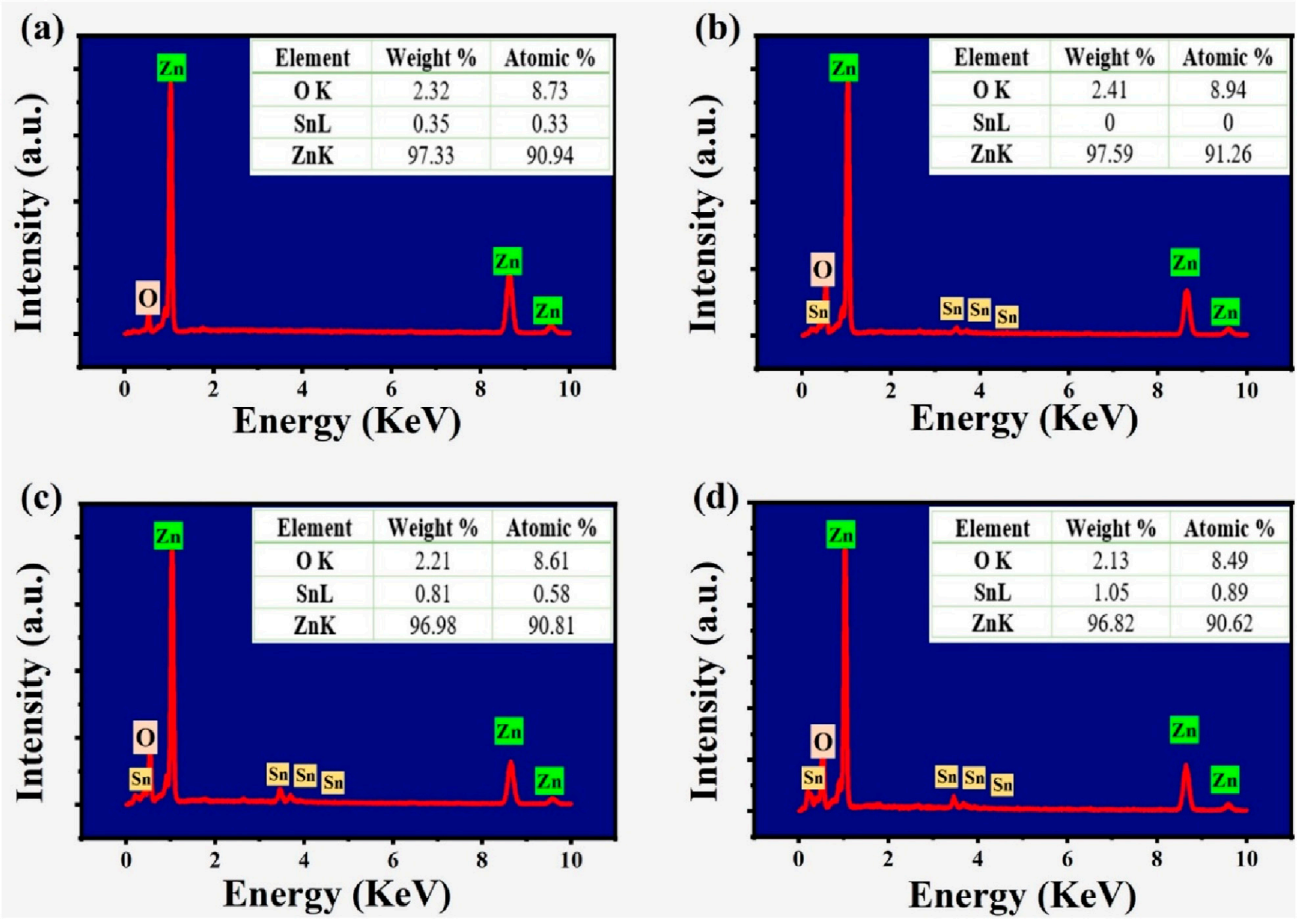
EDX of **(a)** ZnO **(b)** ZS-1 **(c)** ZS-2 **(d)** ZS-3.

Energy dispersive X-ray spectroscopy (EDX) was used to verify the Zn, O, and Sn elemental ratios in the samples. Figure 3 presents the EDX analysis results for ZnO, SZ-2, SZ-2 and SZ-3 samples. The spectra clearly show characteristic peaks corresponding to all elements in the synthesized samples. Undoped ZnO consist of Zn and O as depicted in Figure 3a, while doped ZnO have Zn, O and Sn elements as shown in Figures 3b–d. The measured concentrations of the Sn were found to be in close agreement with the intended values as shown in the Figure insert.

### 3.3 UV-visible spectroscopy

**FIGURE 4:**
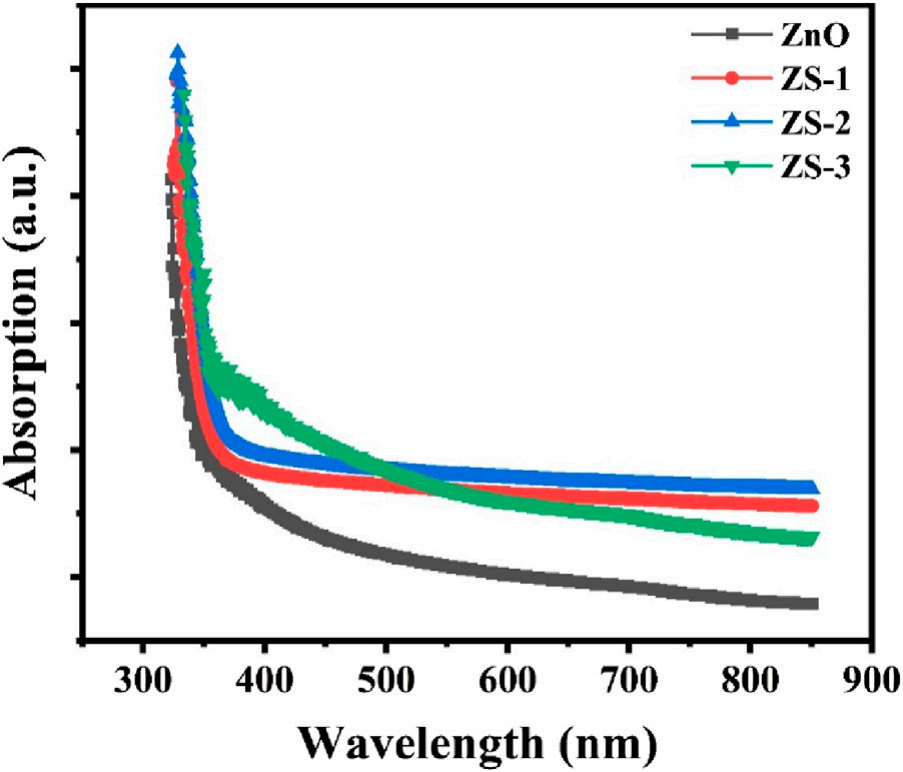
UV-Vis absorption spectra for the Undoped and Sn doped ZnO.

To investigate the optical characteristics of the synthesized samples, UV-Vis spectroscopy was employed. Figure 4 displays the absorption spectra of both undoped and Sn-doped ZnO measured at room temperature across the wavelength range of 250–800 nm. All samples exhibit distinct absorption edges between 350 nm and 400 nm. Compared to undoped ZnO, the Sn-doped samples show noticeably enhanced absorption in this region. This increase in absorbance with doping suggests a modification in the bandgap, which could be attributed to changes in crystallite size and the introduction of lattice distortions. These distortions may arise from interactions between the Sn dopant and the ZnO lattice, including a possible reduction in oxygen vacancies (Safeen et al., 2022a; Ali et al., 2023).

The absorbance spectra show that ZnO has an absorption edge near 357 nm, which is associated with the excitation of electrons from the valence band to the conduction band. When Sn is introduced, the absorption edge shifts toward longer wavelengths a phenomenon known as redshift. This shift can be linked to factors such as increased surface area, the presence of oxygen vacancies, and the substitution of Zn ions with Sn ions in the ZnO crystal lattice (Safeen et al., 2023).

**FIGURE 5:**
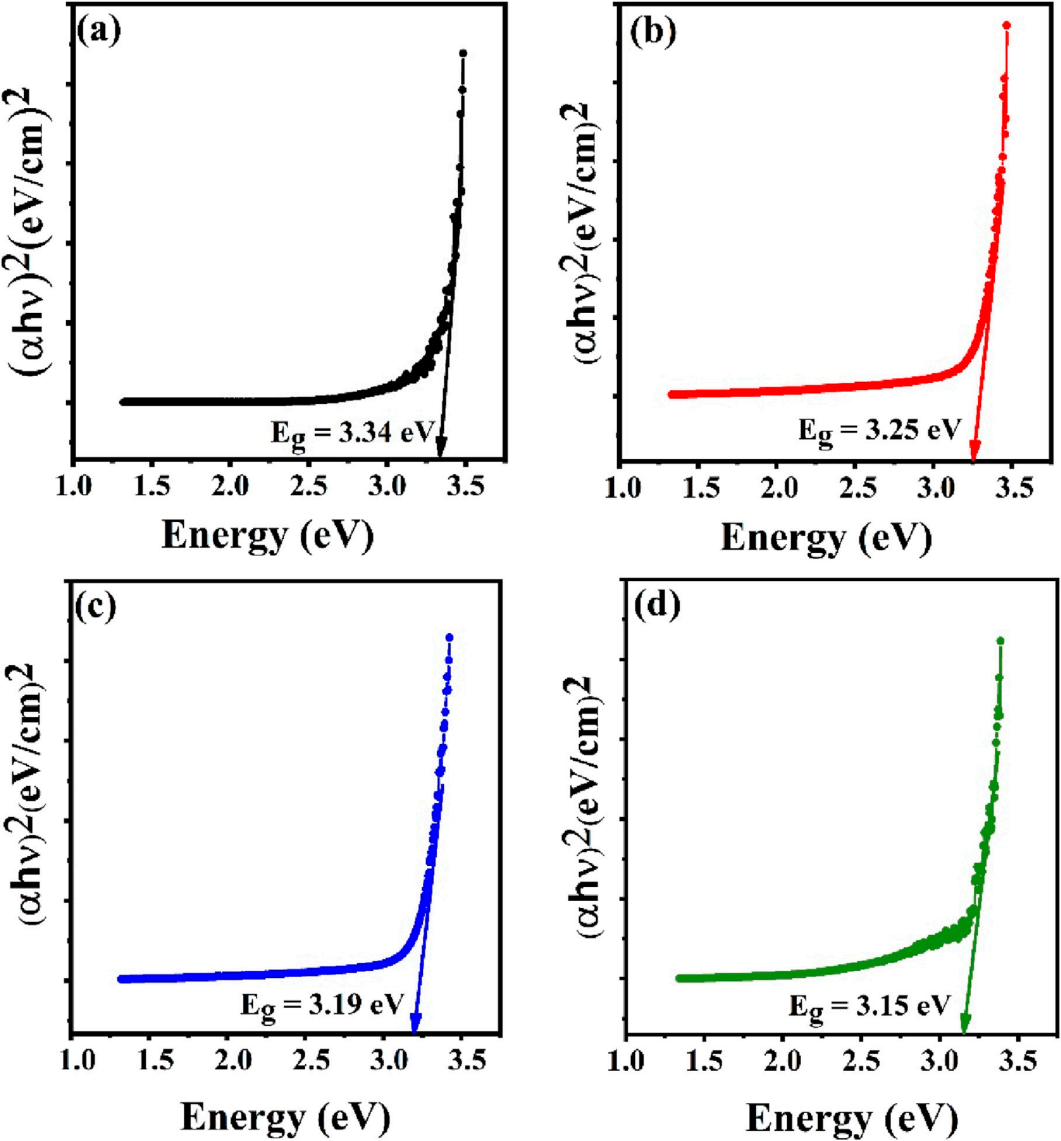
Energy band gap using Tauc plot method for **(a)** Undoped ZnO, **(b)** ZS-1, **(c)** ZS-2 and **(d)** ZS-3.

The optical band gap for the synthesized samples was calculated by Tauc relation (Equation 7) given as;

$$\alpha h\nu =B{\left(h\nu -E_{g}\right)}^{n}$$ (7)

where α is the coefficient of optical absorption, B is a constant and hν is the energy of the incident photon. The value of n is assumed to be 1/2 since ZnO is a semiconductor with a straight bandgap (Safeen et al., 2022b). The energy bandgap was determined by extrapolating the linear region of the (αhν)^2^ versus hν plot to the x-axis. Figure 5 illustrates these Tauc plots for both pure and Sn-doped ZnO nanoparticles. The bandgap of undoped ZnO was found to be 3.34 eV. With increasing Sn doping, the bandgap gradually decreased, reaching a minimum value of 3.15 eV. The reduction in bandgap energy and the enhanced absorbance observed in the Sn-doped ZnO samples may be attributed to the substitution of Zn^2+^ ions with Sn^4+^ ions in the ZnO lattice. This incorporation of Sn^4+^ into the host lattice alters the electronic structure, as reflected by the bandgap shift. Additionally, the observed changes can be linked to quantum confinement effects and a high surface-to-volume ratio. Quantum confinement arises when the particle size approaches the de Broglie wavelength of the charge carriers, causing alterations in the electronic energy levels and resulting in a narrower bandgap (Jia et al., 2024). Moreover, the increased surface area due to the nanoscale size enhances surface interactions, which can further modify the electronic structure and lead to greater light absorption in the doped samples (Mrabet et al., 2015; Ahmed et al., 2014; Gao and Wang, 2024).

### 3.4 Photoluminescence study

**FIGURE 6:**
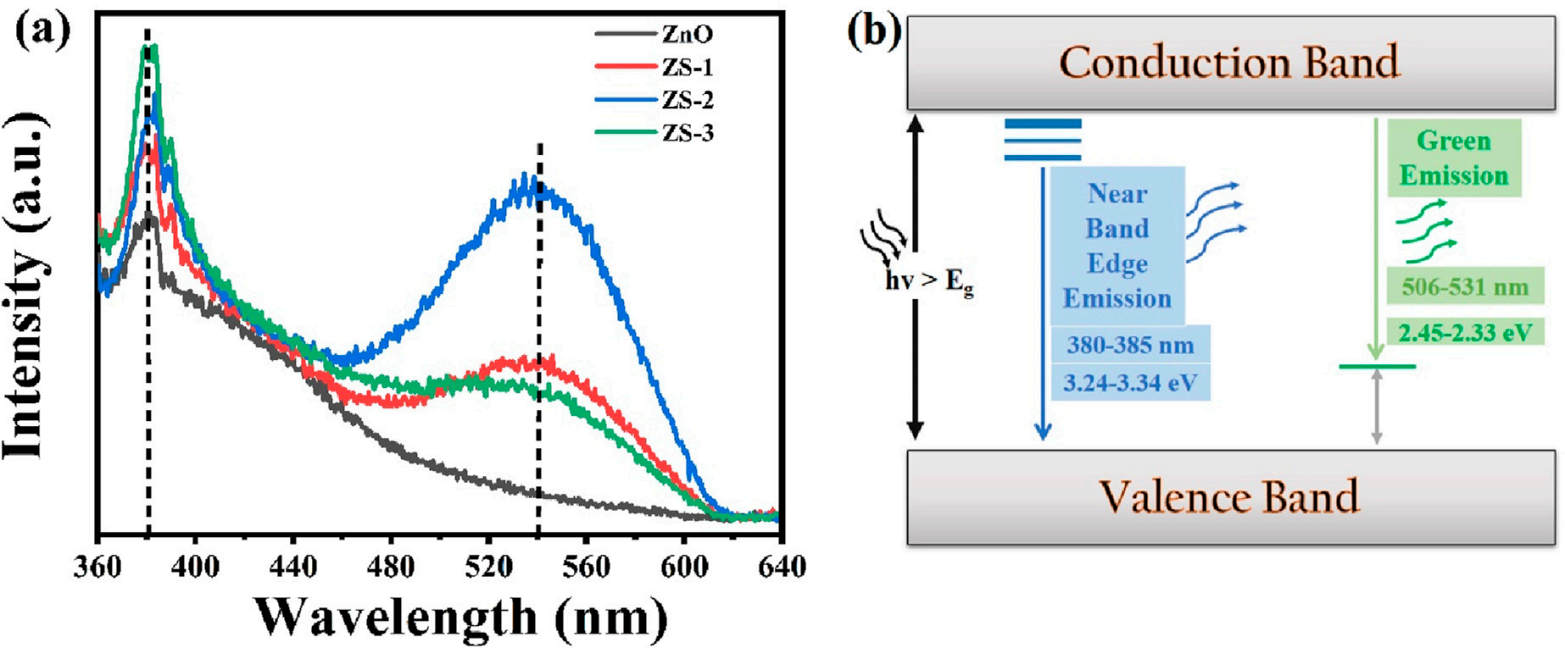
**(a)** PL spectra of pure and Sn doped ZnO, **(b)** Energy band level diagram.

Photoluminescence (PL) spectroscopy is employed to investigate the electronic structure, and discrete energy levels induced by doping of the synthesized samples. The PL spectra of Sn-doped ZnO along with undoped ZnO nanoparticles, recorded over the wavelength range of 350–650 nm, are presented in Figure 6a. All synthesized samples consist of two emission bands in PL spectra, one corresponding to intense UV emission and the other to broad deep-level emission, as shown in Figure 6b. A detailed examination of the PL spectra reveals that both undoped and Sn-doped ZnO samples exhibit prominent peaks in the UV and visible regions. The energy band gap can be inferred from the energy corresponding to the prominent peaks in the PL spectra using the formula E = hν (Hassanien et al., 2021; Huang et al., 2024). In the UV region, emission peaks were observed at around 381 nm for all the synthesized samples. These emissions are attributed to near-band-edge (NBE) transitions, corresponding to band-to-band electronic transitions (Radha et al., 2018). Similar UV emission peaks have been reported in previous studies (Saikia et al., 2015). Notably, the UV emission peak intensity increases with the Sn doping concentration. This behavior aligns with findings in the literature (Hammad et al., 2013). A slight blue shift in the UV emission region was observed, which may be attributed to the Burstein-Moss effect. Conversely, red shifts reported in the literature have been associated with phenomena such as band tailing or exchange interactions. Some distinct peaks were also observed in the visible region around 536 nm corresponding to green emissions which are associated with defects such as zinc interstitial and ionized oxygen vacancies. This emission is a result of the radiative recombination between a photo-generated hole and an electron occupying the oxygen vacancy (Vo) in the lattice (Talam et al., 2012). Usually, Vo vacancies are considered as color centers, which are important point defects in oxides. Oxygen vacancies are widely regarded as the primary defects responsible for the green emission in PL spectra of ZnO. Within the three charge states of oxygen vacancies V_O_, V_O_^+^, and V_O_^++^ the singly ionized oxygen vacancy is considered the main contributor to the green emission (Hussein et al., 2024). Vanheusden et al. proposed that visible emissions are caused by the recombination of electrons in the V_O_^+^ center with photoexcited holes (h^+^) in the valence band (Vanheusden et al., 1996). On the other hand, Wu et al. presented a different mechanism, suggesting that visible emissions arise from the recombination of photoexcited electrons (e^−^) in the conduction band with holes trapped deeply in the V_O_^+^ state (Wu T. et al., 2019). The PL spectra clearly show that the intensity of peaks in the visible region increased by doping, leading to an enhanced concentration of oxygen vacancies. This rise in oxygen vacancies also results in an increase in interstitial Zn. In this study, a clear trend of increasing oxygen vacancies with higher Sn doping concentrations was observed and these defects are primarily for the enhancement of thermoelectric efficiency as discussed in the next section.

### 3.5 Thermoelectric properties

To investigate the thermoelectric properties, the nanopowder samples were compacted into circular pellets under a pressure of 10 tons for 1 minute. To minimize porosity, the pellets were heat-treated at 450°C for 2 hours. Each pellet, with a diameter of 10 mm, was then coated with silver paste to ensure proper electrical contact during the measurements.

#### 3.5.1 Electrical conductivity

**FIGURE 7:**
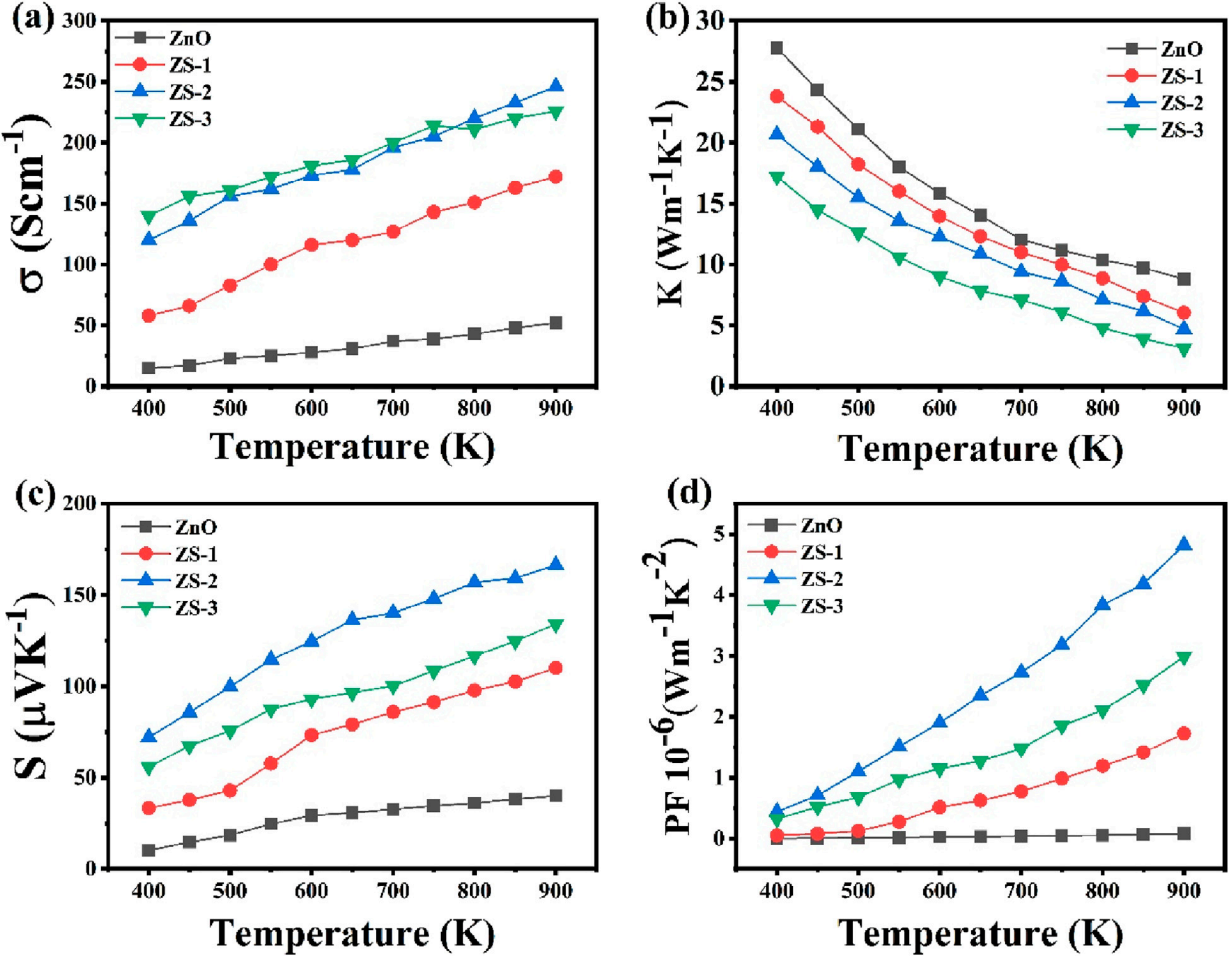
**(a)** Electrical conductivity **(b)** Thermal conductivity **(c)** Seebeck coefficient **(d)** Power factor of undoped ZnO and Sn doped ZnO.

The temperature-dependent electrical conductivity of Sn-doped ZnO samples, along with that of the undoped ZnO sample, is shown in Figure 7a. The electrical conductivity of ZnO increases slightly with rising temperature, while the Sn-doped samples exhibit a more pronounced increase, suggesting semiconducting behavior. It is evident that the presence of Sn significantly influences electrical conductivity. Incorporating small amounts of Sn into ZnO, as observed in the ZS-1 and ZS-2 samples, leads to a notable enhancement in conductivity with temperature. However, for the ZS-3 sample with a higher Sn content, the conductivity gradually decreases above 700 K. At 900 K, the electrical conductivity increases, for undoped ZnO the value of electrical conductivity is 52 Scm^−1^, 172 Scm^−1^ for ZS-1, 246 Scm^−1^ for ZS-2, and decreased to 226 Scm^−1^ for the ZS-3 sample. This trend suggests that the incorporation of suitable Sn doping enhances carrier concentration, thereby enhancing the electrical conductivity. This can be attributed to several competing factors that influence the electrical conductivity such as the introduction of a small amount of Sn, which induces a pronounced donor effect. The substitution of Sn^4+^ ions for Zn^2+^ increases the electron density to maintain charge balance, as the additional electrons act as donors, thereby enhancing electrical conductivity and the addition of Sn^4+^ also results in a reduction in density and grain size, which leads to a higher frequency of scattering events for charge transporters, ultimately reduction electrical conductivity (Young D. et al., 2002; Young D. L. et al., 2002; Hao et al., 2024). Another reason of the enhancement of electrical conductivity is that the substitution of Sn^4+^ ions in ZnO modifies the band structure, introducing an impurity level within the band gap. The observed increase in carrier concentration may also be partially ascribed to band gap narrowing and the suppression of electron-hole recombination with higher doping levels (Lang et al., 2015). It is noteworthy that, as temperature rises, the conductivity of the sample increased, while at higher doping concentration (ZS-3) exhibits a decline. Electrical conductivity is inherently dependent on both carrier concentration and mobility. As the Sn content increases beyond the saturation level (ZS-2), the carrier mobility decreases, leading to a corresponding drop in conductivity. This reduction in mobility becomes the dominant factor, resulting in lower conductivity for the ZS-3 sample. This behavior is likely due to reduced carrier mobility at elevated doping concentrations and relatively high temperatures. The electrical conductivity of the ZS-2 samples investigated in this study was significantly higher than other samples, primarily due to a large number of charge carriers and reduced scattering events associated with their higher density. The decline in mobility at higher temperatures is primarily attributed to enhanced phonon scattering, which disrupts charge transport despite the increased carrier concentration (Chen et al., 2018; Zhu et al., 2025; Roddaro et al., 2014).

#### 3.5.2 Thermal conductivity

Figure 7b shows the thermal conductivity of both undoped ZnO and Sn-doped ZnO across a temperature range of 400–900 K. At lower temperatures, thermal conductivity decreases sharply for all samples, while at higher temperatures, it exhibits an inverse relationship with temperature. In the low-temperature regime, scattering is primarily due to induced charge carriers and static defects. As the temperature increases, phonons with longer mean free paths become more dominant in thermal transport. Phonon scattering is further influenced by various lattice imperfections, including mass fluctuations, structural disorder, dislocations, and grain boundaries, all of which contribute to the reduction in thermal conductivity (Wu Y. et al., 2019; Böer and Pohl, 2023). The substitution of Sn^4+^ ions into the ZnO lattice creates point defects due to the slight mismatch in ionic radii. These defects serve as centers for phonon scattering, increasing lattice disorder and reducing the mean free path of phonons. At elevated temperatures, phonon-phonon scattering becomes the dominant mechanism, further contributing to a significant decrease in thermal conductivity (Jiang, 2019). Sn doping influences the electronic contribution to thermal conductivity by modifying carrier concentration and mobility, increasing electron-phonon interactions, which further hinder thermal transport (Thompson et al., 2015). Defect-induced changes, such as the formation of oxygen vacancies or Zn interstitials, may also contribute to the observed decrease in thermal conductivity. These defects can disrupt the lattice structure, reducing phonon group velocity and, consequently, lowering the thermal conductivity (Biswas et al., 2021). Moreover, Sn doping leads to an increase in grain boundaries, which enhances phonon scattering at these boundaries, further reducing the thermal conductivity. At 900 K, the thermal conductivity decreased from 27 W/mK to approximately 3 W/mK with 1.0 wt% Sn doping. Additionally, the morphological transition from rod-like to more spherical grains with higher Sn doping significantly affects the thermal transport properties of ZnO. In thermoelectric materials, phonon scattering is crucial for reducing thermal conductivity, which in turn enhances the figure of merit (ZT). Spherical or irregularly shaped grains create more grain boundaries and surface defects compared to well-aligned rod-like structures, and these additional interfaces serve as effective phonon scattering centers. This disruption in phonon transport pathways contributes to a reduction in lattice thermal conductivity. The observed change in grain morphology, as seen in our SEM analysis, promotes increased phonon scattering, which aligns with the reduction in thermal conductivity and supports the improvement in the thermoelectric performance of Sn-doped ZnO. Such a decrease in thermal conductivity is beneficial for enhancing thermoelectric efficiency.

#### 3.5.3 Seebeck coefficient

Figure 7c illustrates the Seebeck coefficient as a function of temperature for both undoped and Sn-doped samples. Across the whole temperature range, the Seebeck coefficient remains negative for all samples, suggesting that electrons are primarily charge transporters. Conspicuously, the absolute Seebeck coefficient for Sn-doped samples is greater than that of undoped ZnO. The recorded temperature variation of both the Seebeck coefficient and electrical conductivity deviates from typical behavior. In conventional semiconductors, an increment in carrier concentration generally leads to a reduction in the Seebeck coefficient (Lee et al., 2022). These findings indicate that the conduction mechanism in Sn-doped samples cannot be adequately explained using traditional band theory models but may involve strong electron-electron correlation effects (Terasaki, 2001; Shin and Murayama, 2001). Similar behavior was reported by park et al. for Sn-doped ZnO material (Park et al., 2008). Doping of Sn enhances the Seebeck coefficient, for undoped ZnO 40 μV/K at 900K, 110 μV/K for ZS-1, 166 μV/K for ZS-2 and decreased to 134 μV/K for ZS-3 samples. This improvement at appropriate Sn concentrations is attributed to defects that impede the mobility of low-energy carriers. These defects are likely associated with oxygen vacancies in the lattice structure. A greater number of defects in Sn doped samples as compared to undoped ZnO attribute enhancement in optical absorption and reduction in band gap (Ganesh et al., 2017). Thermoelectric efficiency is greatly influenced by these defects, significantly improving performance. The structural alterations induced by Sn-doping strongly influence the crystal lattice, thereby enhancing the material’s thermoelectric properties (Chen et al., 2020). The observed increase in the Seebeck coefficient and band gap modifications in Sn-doped ZnO are directly linked to defect formation, which is critical for boosting thermoelectric efficiency. Additionally, for samples with higher Sn content ZS-3, the absolute value of the Seebeck coefficient decreases as an increase in Sn^4+^ content. The temperature dependence of the Seebeck coefficient for all the synthesized samples, showing a gradual increase in its absolute value with rising temperature, except the ZS-3 sample.

One possible explanation for the simultaneous increase in electrical conductivity and Seebeck coefficient in Sn-doped samples could be the presence of energy dependent scattering centers or carrier filtering effects. These effects make lower-energy carriers scatter more easily, allowing higher energy carriers more relevant to the Seebeck effect to contribute to electrical conduction. This results in an increase in the Seebeck coefficient while maintaining good electrical conductivity (Jacob et al., 2021; Ghodke et al., 2019). Previous studies have shown that point defects, such as oxygen vacancies, and bulk defects (like secondary phases) can significantly affect both the band structure and transport properties. The enhancement of the Seebeck coefficient with respect to concentration and temperature can be understood through the interplay between the Seebeck coefficient and the scattering factor. In our photoluminescence (PL) findings, oxygen vacancy related zero-dimensional defects likely create additional scattering centers, which improve the scattering factor and thus increase the Seebeck coefficient. These oxygen vacancies may also promote localized states in the electronic structure, which act as scattering centers and filter charge carriers based on their energy (Shi et al., 2020; Li et al., 2010; Wu et al., 2015). When Sn is introduced into the material, it can induce impurity states or resonant levels near the conduction or valence bands. These states are typically sensitive to the valence and electronegativity of the impurity, and when induced by other transition metal dopants, they can be particularly beneficial for enhancing thermopower at middle to high temperatures (Simonson et al., 2011; Jamradloedluk et al., 2024; Rebellon et al., 2024). These resonant states can increase the density of states near the Fermi level, which in turn elevates the Seebeck coefficient. At the same time, they facilitate the movement of charge carriers, maintaining good electrical conductivity (Wu et al., 2017; Zhang et al., 2021).

#### 3.5.4 Power factor

Power factor (PF) of all the synthesized samples is shown in Figure 7d. Sample ZS-2 demonstrates a higher PF than ZnO, ZS-1 and ZS-3. At 900 K, the PF reaches approximately 4.8 × 10^−6^ Wm^−1^K^−2^, which is much higher than other samples. This indicates that an appropriate amount of doping can enhance the electrical transport behavior in ZnO. Consequently, co-doping proves to be a promising strategy for boosting ZnO’s thermoelectric performance.

## 4 Summary

This study explores the structural, morphological, optical, and thermoelectric properties of Sn-doped ZnO nanoparticles synthesized via the hydrothermal method with varying Sn concentrations. X-ray diffraction analysis confirmed the wurtzite crystal structure while SEM analysis revealed a transition from rod-like to spherical and irregular morphologies with increased agglomeration in doped samples. EDX analysis confirmed the successful incorporation of Sn into the ZnO lattice. Photoluminescence spectra exhibited a pronounced green emission, indicating higher defect concentrations with doping. Electrical conductivity improved with Sn doping, and the Seebeck coefficient reached a maximum of 166 μV/K for the SZ-2 sample, surpassing values recorded for other synthesized samples. The Sn-doped ZnO nanostructures analyzed in this study are appropriate for applications in thermoelectric conversion devices as well as to enable the available potential of defects for enhancing thermoelectric performance.

## Data Availability

The raw data supporting the conclusions of this article will be made available by the authors, without undue reservation.
